# Multiple Perspectives on the Adoption of SMART Technologies for Improving Care of Older People: Mixed Methods Study

**DOI:** 10.2196/45492

**Published:** 2024-02-07

**Authors:** Steriani Elavsky, Lenka Knapova, Kamil Janiš, Richard Cimler, Jitka Kuhnova, Tomas Cernicky

**Affiliations:** 1 Department of Human Movement Studies University of Ostrava Ostrava Czech Republic; 2 Anume Ltd Hradec Kralové Czech Republic; 3 Faculty of Science University of Hradec Karlove Hradec Kralove Czech Republic; 4 SeneCura SeniorCentrum HŠH Inc Prague Czech Republic

**Keywords:** adults, older, technologies, technological, caregivers, SMART, mobile phone

## Abstract

**Background:**

Despite the ever-increasing offering of SMART technologies (ie, computer-controlled devices acting intelligently and capable of monitoring, analyzing or reporting), a wide gap exists between the development of new technological innovations and their adoption in everyday care for older adults.

**Objective:**

This study aims to explore the barriers and concerns related to the adoption of SMART technologies among different groups of stakeholders.

**Methods:**

Data from 4 sources were used: semistructured in-person or internet-based interviews with professional caregivers (n=12), structured email interviews with experts in the area of aging (n=9), a web-based survey of older adults (>55 years) attending the Virtual University of the Third Age (n=369), and a case study on the adoption of new technology by an older adult care facility.

**Results:**

Although all stakeholders noted the potential of SMART technologies to improve older adult care, multiple barriers to their adoption were identified. Caregivers perceived older adults as disinterested or incompetent in using technology, reported preferring known strategies over new technologies, and noted own fears of using technology. Experts viewed technologies as essential but expressed concerns about cost, low digital competency of older adults, and lack of support or willingness to implement technologies in older adult care. Older adults reported few concerns overall, but among the mentioned concerns were lack of ability or interest, misuse of data, and limited usefulness (in specific subgroups or situations). In addition, older adults’ ratings of the usefulness of different technologies correlated with their self-rating of digital competency (*r*=0.258; *P*<.001).

**Conclusions:**

Older adults appeared to have more positive views of various technologies than professional caregivers; however, their concerns varied by the type of technology. Lack of competence and lack of support were among the common themes, suggesting that educationally oriented programs for both older adults and their caregivers should be pursued.

## Introduction

### Background

In the Czech Republic, similar to the rest of the world, adults aged ≥65 years belong to the fastest-growing segment of the population, accounting for 19.2% of the Czech population. People aged >80 years belong to the fastest-growing subgroup, and their share among those aged >60 years is expected to increase from 11% to 19% by 2050 [1]. However, with increasing age, the likelihood of comorbidities, functional limitations, and disabilities increases, with negative consequences for self-sufficiency and mobility. The probability of at least partial dependence on the help and care of others or of transition to care facilities increases with age [2], which has implications for the growing demands on the care system in the Czech Republic.

The Czech Republic (together with Poland and Greece) belongs to the European Union countries with the greatest preference for family care in old age [2]. However, although most older adults wish to spend the last years of their lives at home, only a minority fulfill this wish. Although 80% of Czech older adults remain in homecare, where they are primarily cared for by their relatives [3], and 60% wish to live their lives at home, only 21% of deaths in the Czech Republic occur at home [4]. However, preferences for family care decrease with increasing health problems and functional limitations or with the need for specialized care (eg, for chronic diseases or dementia) [2]. Unfortunately, the Czech Republic spends proportionately less on health care and provides only limited support in long-term care compared with other European Union countries [3]. Likewise, the field of private care is not yet fully developed in the Czech Republic, and people are not used to paying extra for care. Apart from family care at home, the state care system for the older adults dominates in the form of either field care services (ie, assistive services or homecare) or state-supported homes for the older adults [5,6].

Modern (SMART) technologies are one of the tools that can be helpful in supporting dignified aging both at home and in the institutional environment. SMART technologies refer to computer-controlled devices that appear to not only act intelligently [7] but also describe technologies capable of monitoring, analyzing, and reporting the status of an object. In the health sciences literature, this topic falls under the eHealth domain. The term “eHealth” is specifically used to describe approaches and devices that promote health or healthy behaviors that use the internet. The narrower field of mobile health refers to the use of mobile handheld devices to support medical and public health practices [8]. eHealth or mobile health technologies also include integrated or connected sensors that provide ways to monitor and improve health (or factors in the environment that affect health), healthy lifestyles, or overall well-being. Technologies specifically developed for older adults or aimed for use by aging populations are referred to as gerontechnology [9].

The application of gerontechnology can help compensate in selected areas of care and support selected needs of older adults [10]. At the same time, informal caregivers can benefit from the use of technology when providing care at home, and technology can help facilitate the care provided in residential or other specialized facilities for older adults [11]. The COVID-19 pandemic further exposed areas in which greater involvement of technology could facilitate care or support the quality of life of older adults [12]. Despite this vast potential, however, a wide gap remains between the innovations and development of new technologies and their adoption by target populations and settings [13-15]. Among the facilitating factors for technology use are motivation, support and feedback received from others, and usability or accessibility, whereas the most commonly cited barriers to the adoption of new technologies are sociodemographic factors (age, education, and skills); personal factors (such as lack of time or other priorities); lack of support; lack of resources or unclear guidance on how to use the technology [16,17]. Different technology acceptance models have also been proposed to help explain the use of technology at the individual level such as the Technology Acceptance Model (see the review by Marangunić and Granić [18]) or the Unified Theory of Acceptance and Use of Technology (see the review by Khechine et al [19]). Although these theoretical models are useful in describing individual differences in technology use or explaining the adoption of a concrete technology, they are not well suited for studying technology adoption in a broader context and describing attitudes toward novel technological advancements. This is particularly true when users lack sufficient familiarity with the technology under investigation, making it challenging for them to express their attitudes or assess predictor variables due to their limited experience or knowledge.

### Objectives

In this study, we focused on evaluating the potential of SMART technologies in the care of older adults. Specifically, we focused on multiple perspectives on the adoption of SMART technologies in care for older adults. We concentrated on inputs from different groups of stakeholders. First, we interviewed professional caregivers from a variety of settings providing care to older adults. Second, we conducted a cross-sectional web-based survey with adults attending the Virtual University of the Third Age (V-U3A). Third, we interviewed experts on aging on their views. Finally, we included a case study focusing on the collaboration between a university start-up company and a private care provider with respect to the adoption of a specific technology, the ANUME smart bed system. The overarching research questions were as follows:

1. What are the barriers to the adoption of SMART technologies in the care of older adults?
2. How can these barriers be overcome?

## Methods

### Overall Conceptual Framework

To effectively represent the spectrum of applications in which the use of technology intersects older adult populations, in this study, we applied a triangulation method, combining quantitative (cross-sectional) and qualitative data from several sources [20,21]. The selection of target groups and type of data used reflected several overlapping (and perhaps underrepresented) realities. First, there is an interplay among technology use, perceived competence, and attitudes or motivation in using technology, especially among older adults or those who perceive themselves as generally less technologically savvy [22]. Despite the general contemporary increase in the use of information and communications technologies across all age groups, in the context of the Czech Republic, the use of technologies by older adults remains low [6,7]. This makes it challenging to effectively evaluate the attitudes toward technologies among this target population, as they lack the necessary frame of self-reference (ie, experience with said technologies) to do so. Relatedly, the probability of at least a partial dependence on the help of caregivers or of a transition to care facilities increases with age [2], making it likely that most older adults will be cared for through external resources (ie, family members and professionals). As such, caregivers thus represent the target market for care-related technology or operate as advocates recommending the technology to other users, including older adults themselves or those caring for them. Simultaneously, they bear the heavy burden associated with caring for an aging person (often being individuals with limited function and autonomy) and are therefore positioned to benefit the most from using technology as part of the care they provide. In light of these complex intersections between technology use and aging, and in an effort to more effectively examine those with an advanced aptitude toward technology in this framework of relations, we chose to focus on two primary target groups: (1) people who care for older adults in a variety of settings and (2) older adults who are also attendees of the V-U3A (ie, representing more technologically “savvy” older adults who are the most likely users of SMART technologies). To supplement the views of these care providers and older adults themselves, we also used insights from an invited group of experts in the field of aging, with the belief that their perspectives would help contextualize the experiences of care workers and older adults in a broader context. Finally, to illuminate the dialogic processes that comprise the research-application continuum, we have included the details surrounding a concrete example of the adoption of technology in older adult care. Concretely, we have provided a case study of implementation that further highlights the “real-world” application process by recounting the process of cooperation between researchers, a commercial entity (a university-based start-up company), and an application partner (the SeneCura company) that provided continuous feedback on the partial and evolving functionalities of the specific technology (ANUME smart bed), communicating both the specific expectations of future users (eg, in terms of function, operation, and safety) and the subsequent experience of technology adoption, underscoring the ways in which intention and application are always in process.

### Study 1: Perspectives of Professional Caregivers

#### Sample and Recruitment

We conducted semistructured interviews with 12 professional care providers: 5 (42%) workers from older adult homes, 3 (25%) from nursing services, 3 (25%) from social services, and 1 (8%) from a medical facility. Recruitment was performed through selected cooperating facilities and through chain referral using purposive sampling strategy (Multimedia Appendix 1).

#### Procedure

Conducted by the study coauthor KJ, a Doctor of Philosophy researcher (male) with extensive experience in qualitative methods and the area of aging, the interviews were performed from September 2020 to March 2021, that is, during the period of the COVID-19 pandemic, which complicated recruitment efforts to some extent and affected the form of the interviews (some interviews took place face to face, some were internet-based via Zoom [Zoom Video Communications, Inc] or Skype [Skype Technologies]). All respondents provided informed consent and gave permission for the interviews to be audio or video recorded for the purpose of obtaining verbatim transcripts of the interviews to be analyzed.

#### Measures

The interviews followed a semistructured “interview guide” (Multimedia Appendix 2).

### Study 2: Perspectives of Older Adults

#### Sample and Recruitment

To examine the perspective of older adults, we conducted a web-based cross-sectional survey with adults aged ≥50 years. The recruitment strategy focused on students at the V-U3A, which is organized by the Faculty of Business Economics of the Czech University of Life Sciences in Prague. The V-U3A allows interested parties from all over the Czech Republic to pursue higher education at V-U3A as part of an adult continuing education (attendees must be Czech citizens of retirement age). Education within the V-U3A takes place through a combination of internet-based and collective “in-person” methods of education. The listeners meet once every 14 days in a so-called consultation center (mostly a city or municipal office, library, school, or older adults’ club in different cities or towns), where they have access to a PC with an internet connection, among other things. As a target group, V-U3A listeners therefore included older adults with at least basic digital literacy and an assumed ability to handle participation in a web-based questionnaire survey. The focus on V-U3A listeners was also partly related to the complicated epidemiological situation when there was no face-to-face teaching, and most of the events aimed at the target group of older adults did not take place in person.

#### Procedure

Respondents were contacted via a mass email, which was distributed upon request by the relevant coordinators in individual regions of the Czech Republic (4 coordination centers were randomly selected from each region and were invited to participate). The recruitment email described the study and contained a link to the web-based questionnaire. Informed consent was presented as the first page of the survey, and subsequent completion of the survey indicated consent with participation. Data were collected between January 2021 and April 2022.

#### Measures

The survey was conducted on the web using the Qualtrics platform (**Qualtrics** International Inc). It included basic sociodemographic questions and assessed the level of digital literacy with a single item “How would you rate your skills in using IT technologies (eg, computer, smartphone, tablet)?” rated on a scale from 1 (“beginner”) to 7 (“expert”). The respondents then rated 21 different technologies or SMART devices marketed commercially to older adults and their caregivers. The evaluated smart technologies included technologies that can be used to satisfy various needs of older adults or caregivers (related to everyday practical tasks, social and emotional support, health monitoring or managing, and compensatory assistance rehabilitation) [23] and spanned the entire spectrum of technologies from simple ones (such as smart thermometer) to complex smart technologies (internet-based assistants and smart home systems). The individual technologies and the purpose of their use were first briefly described (together with a photo), and the respondents then evaluated the extent to which they found them useful (on a scale of 1 [“minimally useful”] to 10 [“maximum useful”] with the option to choose “don’t know”). For each technology, the respondents had the opportunity to describe their concerns in an open answer using the given technology. The questionnaire also included questions for caregivers who care for older adults aged >65 years. The survey was conducted anonymously.

### Study 3: Perspectives of Experts in the Area of Aging

#### Sample and Recruitment

Email interviews were conducted with experts in the area of aging including researchers, clinicians, and aging business or nonprofit representatives. The goal was to incorporate perspectives of various stakeholders, including but not limited to academic researchers. Participants were selected based on convenience and prominence in the aging sector in the Czech Republic across the fields.

#### Procedure

A total of 28 aging experts were contacted via email by the research project principal investigator in 2 waves during the summer and fall of 2021: 11 during the first wave in July 2021, followed by 18 in the second wave in September 2021. A reminder email was sent 5 to 8 weeks after the first invitation. The email invitation included a file containing the survey questions to be returned to the researchers via email. Of the 28 contacted experts, 15 (54%) were primarily academic researchers, 7 (25%) were representatives of older adult or aging organizations, and 6 (21%) were clinicians. Participants provided textual answers to 5 open-ended questions. The answers were of varying length, detail, and comprehensiveness, as no guidelines on the detail and extent of the answers were provided by the researchers.

#### Measures

With respect to time constraints of the respondents, the email interview was constructed to be brief with 5 open-ended questions on SMART or modern technology for the older adults. The questions covered the following areas: (1) opportunities of SMART or modern technologies for older adults, (2) barriers to the adoption of SMART or modern technologies for older adults, (3) good practice examples, (4) technologies that should be commonplace in older adult care, and (5) recommendations to stakeholders researching and implementing technologies for older adults (Multimedia Appendix 2).

### Case Study: Perspective of Care Providers

#### Sample and Recruitment

For the case study, SeneCura SeniorCentrum Hradec Králové, located in Hradec Králové, Czech Republic, entered the project as an application partner on the grant project application along with the University of Hradec Králové start-up company ANUME Ltd. The SeneCura SeniorCentrum is a modern private residential facility for social services offering approximately 150 beds. The facility provides the following 2 services in terms of the Social Services Act: home for older adults and home with a special regime. Most of the clients of the home with a special regime have dementia, most commonly Alzheimer disease. Furthermore, most clients are polymorbid and require 24-hour care. SeneCura currently operates 15 similar facilities in the Czech Republic, with a total capacity of approximately 2100 beds. The ANUME smart bed product (developed by DeepLab Ltd., and later renamed to ANUME, Ltd., a second application partner in the project) consists of pads measuring the microvibrations of the human body, which are placed under the mattresses in the clients’ bed. These pads can be used to determine the presence of the person on the bed and the elapsed time since the last positioning or repositioning, and thanks to special algorithms, the pulse and breathing frequency of the person lying on the bed can also be obtained from the microvibrations. Nursing staff have tablets on which this information is displayed, and problematic conditions are notified through visual and audio alerts.

#### Procedure

Cooperation with the application partner included regular meetings every 14 days, with group discussions based on the staff feedback and fine-tuning to meet the specific needs of care providers. Two of these meetings were in the format of a half-day “workshop” (July 2 to September 23, 2020), where there was an in-depth debate on the specific functionalities of the ANUME smart bed system. Initially, 10 ANUME smart bed systems were placed in the SeneCura care facility, where regular consultations and tuning of the product took place across 12 months. Later, 5 additional ANUME smart bed systems were placed in 5 homes where family members cared for older adults.

#### Measures

The process of implementation included interviews with nursing staff, clients, and management to ensure that the product effectively targeted everyone’s needs. The main outputs from the interactions were summarized and agreed upon by all stakeholders before proceeding with system adjustments.

### Data Analysis

Data from study 1 (in-person interviews) and open-ended responses from the web-based survey in study 2 were analyzed using content analysis and group interpretation. Each interview was read by 2 independent people (SE, the researcher, read all interviews; LK and KJ read 6 interviews each). During the reading, each researcher took notes in their Excel (Microsoft Corp) file, which was structured to align with the “interview guide.” This was followed by a group discussion and interpretation of the most important themes. For analysis of the open-ended responses in study 2, the data were coded by 3 independent coders based on a predetermined coding scheme. The coding scheme was developed based on a preliminary content analysis of 50% of the data by the lead study author (SE) and refined in the process of subsequent coding. Interrater reliability was evaluated on randomly selected data that were coded by all 3 coders (about 10%) and ranged from 0.42 to 0.99 for the checked categories. Inconsistencies in coding were then discussed among the 3 coders and the first study author and resolved them after clarification and upon mutual agreement. This process was repeated 3 times to increase the precision and consistency of coding throughout the coding process. Frequency analysis on the coded data was performed. In addition, quantitative analysis of web-based survey data in study 2 included basic descriptive statistics and Pearson correlation coefficient calculated using SPSS (IBM Corp). Missing data were handled through listwise deletion. For study 3, due to the varying length of the answers in the email interviews and the inability of the researchers to elicit further information on the topic, no advanced qualitative data analysis (such as thematic analysis) was used. Individual answers to each of the 5 questions were merged and subsequently synthesized by the researchers. For the case study, we summarized the near 2-year experience of a 3-way collaboration between a start-up technology company, private care provider, and academic researchers.

### Ethics Approval

The project and study procedures were approved by the Ethics Committee at the University of Ostrava (application number: OU-78256/90-2020).

## Results

### Perspective of Professional Caregivers

#### Sample Description

The average age of the interviewed professional caregivers was 44 (SD 9.2; range 27-56) years. Most of the interviewees (8/12, 67%) had a university education, 25% (3/12) had a high school diploma, and 8% (1/12) had vocational training without a high school diploma. The respondents had been in their current job for an average of 12 (SD 9; range 0.5-30) years, and 7 respondents had previous relevant work experience with older adults before working in their current positions. The average length of the interview was 41 (SD 10.5; range 27-63) minutes.

#### Findings

On the basis of the caregivers’ reports, they used a limited number of technologies in their work. The primary method of documentation was paper and pencil. Nursing home managers and social workers mentioned the use of a computer with a specialized software application for documenting care, and some care workers mentioned the use of readers to record care activities (even in these cases, however, they duplicated the records by using the “paper-pencil” method if the reader failed). Other “tools” mentioned included the use of a weighing chair, monitoring bracelets with alarms at the entrance (all used in one specific older adult home), and an interactive table. Respondents also mentioned that they used mobile phones for work and that their activization workers used tablets when working with clients. In these cases, the clients were passive users of tablets or phones, as the activization worker would search for music or photos and then share them with the clients. Tablets were also used to communicate with family (mentioned by 1 worker in an older adult home).

When discussing the specific technologies, although several are already commonly available, their penetration into common practice in care for older adults was seen as problematic. This was partly related to the technologies being perceived as costly and out of reach for a Czech older adult or in the context of “typical” care provided. However, the main problem that was identified was the social service care workers’ perceived rigidity to change:

It’s just that everyone here is like that, I don’t know, like that, they’re just slow to open to these technologies and just see the problem behind everything, yeah [...]; [...]that if somewhere I don’t know something new electronic, the workers in social services are terribly irritated by it, that it will be extra work again

...

Although the caregivers saw the advantages of a particular technology that would facilitate some of the tasks that they were performing or help satisfy the needs of older adults, they were not willing to consider including it in their work routine. Existing procedures and approaches were assessed as sufficient and easier as they were used to them. This attitude was also partially supported by the caregivers’ assessment of their clients’ digital literacy or their own negative relationship with technology. Caregivers presumed the digital literacy of older adults to be low, or they expected that physical and other limitations would prevent older adults from using them:

You know what, it’s just that I can’t, like, when I drive around these families and the elderly, they don’t have any modern technology at home, yes, you can see an old television, for example, and I can’t imagine that he would understand this, control it like and that is not to demean them, please, yes, but I don’t know how they would at all with such modern technology, well, I don’t know.

The interviews also showed that the caregivers would be reluctant to implement some smart technologies in their work even if they could, especially when it came to technologies that were perceived as “displacing” older adult needs. A typical example was the perception of a social robot as a replacement for personal contact and not as a supplementary technology aimed at increasing socialization and entertainment at moments when personal contact might be scarce. The following was one respondent’s reaction to the usability of the interactive robot:

[...] Like, good, I would be afraid of that too, I wouldn’t want it either, like. Although it looks, like, peaceful. But I, I would be afraid of that [...].

Additional themes that emerged in the conversations included the lack of trust of old people in anything new, including technology and privacy concerns in general (eg, when it comes to constant surveillance in the case of motion detection systems); specifics of use (control, charging, etc) and the expected problems associated with them; the need for simple control or operation not only when used by the older adults themselves but also by the caring staff; and differences in the perception of technology by the type of care facility (small and family-oriented facilities perceived to have a sufficient number of workers to provide quality care even without technology solutions) or depending on the condition or diagnosis of the client (eg, for clients with cognitive deficits or dementia, any “wearable” technology was perceived as useless and not helpful).

Despite the identification of many barriers or nuances in the use of technology, there were positive comments that underlined the potential of technology to improve the quality of life of older adults and reduce the workload of the care staff:

Exactly, and it will also make life easier for the seniors, like the feeling that I am able to do something for myself again, I go back to that spoon, to eat soup, that is so important for them, but so terribly important, they just here they will fully appreciate such a step with a smile, with enthusiasm, that it will be better again, that they will simply take a step forward again, and that is just great.

### Perspective of Older Adults

#### Sample Description

In total, 521 respondents clicked on the survey link. Of them, 145 (27.8%) respondents were excluded: 60 (41.4%) respondents did not answer a single question, 60 (41.4%) answered one question but did not complete the ratings of technologies, and 25 (17.2%) did not provide key sociodemographic information (age). Of the remaining 376 respondents, 7 (1.9%) stated that they were aged <50 years and were excluded. Of the remaining 369 in the final sample, 58 (15.7%) had some missing data: 2 (3%) <1% missing; 5 (9%) <20%; 7 (12%) <50%; 6 (10%) <75%; 38 (66%) ≥75%. The final sample of adults consisted of 369 respondents aged >55 (mean 71.1, SD 5.4; range 57-95) years, with a completion rate of 65%. Most respondents (n=313, 84.8%) were female, and most (n=355, 96.2%) were retired. Most respondents reported having secondary education with (n=186, 50.4%) or without a diploma (n=21, 5.9%), and 33.3% (n=123) had a university degree. The respondents (n=259, 70.2%) rated their economic situation as average. Their perceived digital competence varied and rated on average 3.67 (SD 1.01) on a scale from 1 (beginner) to 7 (expert). Moreover, 16% (n=59) of the respondents said that they provided care to someone aged >65 years. Therefore, these respondents were able to provide the perspective of a caregiver in addition to the perspective of older adults. Descriptive characteristics of the sample are depicted in Table 1.

**Table 1:** Description of survey respondents (n=369).

Variable			Values, n (%)
**Age (years)**			
	55-59	2 (0.5)	
	60-64	24 (6.5)	
	65-69	138 (37.4)	
	70-74	111 (30.1)	
	75-79	65 (17.6)	
	80-84	24 (6.5)	
	≥85	5 (1.4)	
**Gender**			
	Female	313 (84.8)	
	Male	55 (14.9)	
	Missing or not reported	1 (0.3)	
**Marital status**			
	Single	6 (1.6)	
	Married	206 (55.8)	
	Partner relationship	12 (3.3)	
	Divorced	50 (13.6)	
	Widowed	95 (25.7)	
**Education**			
	Elementary (including unfinished)	7 (1.9)	
	Vocational training without diploma	21 (5.7)	
	High school education with diploma	186 (50.4)	
	Higher education	28 (7.6)	
	University education	123 (33.3)	
	Missing or not reported	4 (1.1)	
**Employment status**			
	Retired	355 (96.2)	
	Employed	15 (4.1)	
	Private business	6 (1.6)	
	In household	2 (0.5)	
	Full-time caregiver	3 (0.8)	
	Other	8 (2.2)	
**Self-rated economic situation**			
	Below average	6 (1.6)	
	Rather below average	32 (8.7)	
	Average	259 (70.2)	
	Rather above average	56 (15.2)	
	Above average	5 (1.4)	
	Missing or not reported	11 (3)	
**Self-rated digital skills**			
	1 (beginner)	10 (2.7)	
	2	33 (8.9)	
	3	113 (30.6)	
	4	124 (33.6)	
	5	63 (17.1)	
	6	13 (3.5)	
	7 (expert)	1 (0.3)	
	Missing or not reported	12 (3.3)	

#### Findings

Among the 3 highest-rated technologies with respect to their usefulness for older adult care were wearable fall detection sensors (SOS buttons for calling for help; mean 8.9, SD 1.8), tablets (mean 8.8, SD 1.8), and smartphones (mean 8.7, SD 2.0). Among the lowest-rated technologies were interactive robots (mean 4.5, SD 2.9), followed by virtual reality (mean 6.2, SD 3.0) and smart alarm clock (mean 6.3, SD 2.8). The average rating across all technologies (mean 7.4, SD 1.5) was positively related to self-rated information and communications technology abilities (mean 3.7, SD 1.1; *r*=0.238; *P*<.001).

Overall, the older adults had no or few concerns about the technologies, with 69.8% of the respondents stating that they had no concerns when averaged across the technologies. The most frequently mentioned concerns across technologies were as follows: certain technologies seemed as unnecessary, redundant, or suitable only for certain subgroups of older adults (eg, those with health problems or dependent on the care of others), the fear of loss of privacy or misuse of the acquired data, the inability or unwillingness of the older population to learn how to handle new technologies or fully use all their functionalities, or worries about making mistakes when using the technology. However, many cited advantages of various technologies, commenting that they had personal experience with them and considered some to be essential (eg, smartphones). Detailed results are presented in Table 2 and Multimedia Appendix 2.

**Table 2:** Ratings of technologies by survey respondents (n=369).

	Participants, n (%)	Values, mean (SD)
Fall detector	310 (84)	8.88 (1.75)
Tablet	314 (85.1)	8.78 (1.77)
Smartphone	313 (84.8)	8.73 (2.05)
Smart bed	291 (78.9)	8.65 (1.98)
Smart contactless thermometer	301 (81.6)	8.59 (1.96)
Smart spoon	295 (79.9)	8.42 (2.21)
Interactive desk	297 (80.5)	8.07 (2.18)
Smart pill dispenser	290 (78.6)	7.95 (2.46)
Smart lightbulb	300 (81.3)	7.87 (2.45)
Witrack	276 (74.8)	7.83 (2.41)
Environmental sensors	283 (76.7)	7.77 (2.5)
Mean usefulness across technologies	334 (90.5)	7.43 (1.54)
Mobile apps	226 (61.2)	7.11 (2.79)
Fitness bracelet	305 (82.7)	7.10 (2.68)
Virtual assistants	244 (66.1)	7.04 (2.66)
Smart cup	284 (77)	6.69 (2.91)
Smart scale	292 (79.1)	6.65 (2.77)
RFID^a^ chip	301 (81.6)	6.5 (2.62)
Barcode reader	285 (77.2)	6.36 (2.86)
Smart alarm clock	282 (76.4)	6.29 (2.78)
Virtual reality	260 (70.5)	6.2 (2.98)
Interactive robots	238 (64.5)	4.55 (2.91)

^a^RFID: radio frequency identification.

### Perspective of Aging Experts

#### Sample Description

A total number of 12 experts responded to the invitation by October 2021. However, 3 of the responses did not include any answers to the survey: 1 participant reported not working in a relevant field, 1 participant was worried about sharing their “know how,” and 1 participant was only interested in “serious collaboration.” A total of 9 experts provided answers to the survey questions. Of them, 8 (89%) were female participants, and 1 (11%) was a male participant; 7 (78%) were academic researchers, 1 (11%) was a clinician, 1 (11%) was a representative of an older adult or aging organization. Overall, the response rate was approximately 50% for academic researchers and as low as 15% for clinicians and representatives of older adult organizations, which have proven to be very difficult-to-reach populations.

#### Findings

There was an agreement among the experts that SMART technologies can bring about large benefits to older adults, especially in terms of increasing or prolonging self-sufficiency and autonomy (eg, through the use of assistive technology and web-based cognitive rehabilitation) and increasing quality of life and decreasing loneliness (eg, through web-based communication tools). Respondents also mentioned that the use of SMART technologies could lead to improved and more efficient care for the older adults and decreased caregiver burden through remote monitoring, shared or distributed responsibilities among caregivers, or web-based rehabilitation options. In addition, several respondents highlighted the facilitation of communication and interaction via technology.

However, a series of barriers to the use of SMART technologies for older adult care was perceived. These could be grouped into (1) obstacles on the side of older adults, (2) objective barriers, and (3) lack of a facilitator. On the side of older adults, respondents mentioned low digital literacy, fear, and negative attitudes, as well as declined cognitive abilities (eg, learning and memory). Objective barriers that were voiced were high costs, nonavailability of SMART technologies to all older adults in all areas, and technologies that were too complex and not customized for their needs. Importantly, respondents mentioned the lack of a facilitator who would introduce the technology to older adults, provide support in case of technical issues, and serve as an intermediary to caregivers or clinicians. The following quotes illustrate this issue:

Furthermore. The absence of experts[...] who could convey information about possible technologies. Their benefits and limits. Social workers play a key role in the introduction of technology for the elderly and especially in their support.

There is a lack of specifically “senior” help desks and people who would physically come and look at the computer problem. so that the senior is not dependent only on the children.

When asked to provide examples of good practice of the use of SMART technologies, some experts mentioned specific technologies, whereas others mentioned systemic practices aimed at implementing technologies for older adult care. In particular, a (social) system that enables older adults to live autonomously at home for as long as possible as well as educates caregivers and social workers about technology was envisioned. Specific technologies perceived as “good practice” ranged from security or monitoring systems and SOS buttons through web-based communication tools to robots for various purposes such as socialization and help with personal hygiene.

Similarly, the level of complexity and technological advancement varied in the experts’ views on technologies that should be commonplace in older adult care. The recommended technologies varied from personal hygiene robots, voice-operated smart beds, or technology-enhanced cognitive rehabilitation, and smart tools of daily living to SOS buttons, movement detectors, and monitors of health indicators. In addition, web-based communication tools including telemedicine were often mentioned.

Finally, the experts provided recommendations to stakeholders researching and implementing technologies for older adults. There was an agreement on the need to consider the whole spectrum of older adults with varied cognitive and physical abilities and contexts and to consider the specifics of this group with relation to technology (eg, insecurity and need to preserve dignity). Importantly, they stressed the need to develop the technologies directly for older adults according to their needs. Technologies should then be tested with all involved parties: older adults, experts, and caregivers. One expert summarized this as follows:

Show them what you can do and get advice on what you should be able to do...

### Perspective of Care Providers

#### Case Description

The main motivation of the application partner, SeneCura, was the use of SMART technology to reduce the care burden of their workers and improve the quality of care in their facility. The cooperation was established based on a specific product, the ANUME smart bed developed by ANUME Ltd.; however, the application partner also showed interest in other relevant technologies that could provide solutions for additional needs (eg, temperature-monitoring solutions and GPS location or detection for leaving the “safe” zone of the device).

The main target groups were social workers and nurses who would use the information provided by the ANUME smart bed. Targeting caregivers in direct care was not a priority and was also perceived as potentially problematic due to the frequent turnover of staff members and the perceived limited ability of the staff (who are often foreign workers) to competently handle technology. During the implementation process, SeneCura identified an additional target group, namely, physicians who take over the care of the older adults in case of transfer to hospital, emergency, or specialized care.

#### Findings

The collaboration between the researchers, start-up ANUME Ltd., and the SeneCura care facility led to continuous improvement of the ANUME smart bed product. Feedback received from the staff and facility management led to both hardware and software adjustments as well as continuous improvement of the application that displayed the monitored data. For example, the original intention was to install only the individual sensors under the mattress and place the computing and communication unit together with the display on the bed frame, which was sufficient in terms of functionality, but the problem was the need to be able to “disinfect” the sensors (despite not being in direct contact with the client but under the mattress) and possible repositioning of the sensors. This need was also intensified during the COVID-19 pandemic. Feedback from the nursing staff also revealed the uselessness of a display placed directly on the bed (staff expressed that they were not using it and the light emitted, even in subdued mode, was bothering some clients). The display was later removed based on this feedback and for economic reasons. The most suitable way to place ANUME in beds proved to be embedding the sensors and all the necessary elements of the system, that is, the sensors themselves and the computing and communication module, in a thin pad. This pad is made of washable material that is intended for use in a hospital environment and is fully disinfectable. It is created from a material that allows the mattress to breathe despite being waterproof. In addition, the user interface underwent changes from a stand-alone bedside monitor to an internet-based web interface. Finally, the tablet application was continuously modified based on the staff and management feedback. As a result of these modifications, the management began to make extensive use of positioning reports (generated based on ANUME system data), which allowed for monitoring of client positioning or the lack thereof. At present, management of the SeneCura facility regularly uses positioning incident reports to evaluate the quality of care of individual shifts and at higher levels of management to compare individual workplaces. At the staff level, night shifts especially appreciated the constant overview of the presence of clients in bed and the staff’s ability to respond in time when the system alerted them that the client had left. The system settings allow adjustment of the alarm schedule to, for example, not alert during a shorter stay out of bed such as a visit to the toilet, but alert in the case of a longer stay, which may be indicative of a risk situation such as a fall.

## Discussion

### Principal Findings

**Figure 1:**
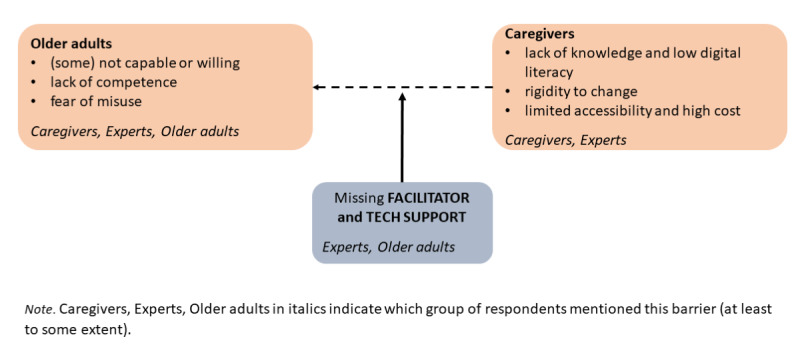
Conceptual representation of barriers to the adoption of SMART technologies from different perspectives. Note: caregivers, experts, and older adults in italics indicate which group of respondents mentioned this barrier (at least to some extent).

This study used mixed methods and multiple sources of data to examine the potential of SMART technologies to improve care for older adults, identify key barriers to their adoption, and demonstrate how barriers can be overcome to facilitate the use of technology in caregiving contexts. Perspectives from different groups of stakeholders (ie, professional caregivers, older adults, and aging experts) and a concrete example of the implementation of a specific type of technology have demonstrated that despite the potential SMART technologies offer for improving older adult care, multiple barriers persist to their broader adoption. The key barriers were perceived to be the inability or unwillingness of at least some older adults to use SMART technology, partly due to low perceived competence and fear of misuse of the data it provides (mentioned by all stakeholders). On the side of the caregivers, a lack of knowledge about technologies, low digital literacy, rigidity to change, and inaccessibility were identified as important barriers by both caregivers and aging experts. Older adults and aging experts agreed that a potentially useful way to overcoming these barriers could be the inclusion of a facilitator or tech mediation worker who would provide support not only during the initial adoption phase but also throughout the use of the technology (the main themes are represented in Figure 1). In addition, the presented case study indicated that meaningful improvements in technology development can be achieved and new opportunities for the involvement of new technologies can be identified through a collaborative process involving all stakeholders, thus enhancing the potential for technology adoption.

### Comparison With Prior Work

Previous research has shown that age plays an important role in the use and adoption of new technologies [24-27]. Although older adults use new technologies less and perceive more barriers to their use, the use of new technologies is also related to the level of education [25-28]. Research also shows that older adults are able and willing to use new technologies if they find them useful and simple enough to use [18]. Thus, we should not approach older adults as a monolithic group and anticipate reluctance or fear of interacting with technology without offering opportunities for older adults to try new technologies and proper support in the process of their adoption (eg, through caregivers or relatives who can introduce technology to older adults and teach them to work with it) [29].

It was evident from the responses by older adults that they found SMART technologies highly useful (7.4 on a 0-10 scale), and they mentioned few concerns in relation to the different SMART technologies surveyed. Among the specifically mentioned concerns were fear of loss of privacy or data abuse, which can be considered as relatively universal concerns, and although they are prevalent, they do not typically lead to abandonment of technologies [30]. Views of the older adults also indicated that various SMART technologies might be perceived very differently not only based on their type and function but also possibly the amount of exposure that older adults have had to the given technology and their related understanding of its functionality. For some technologies, older adults did not see the point of using them personally and rather found them useful for specific populations “in need;” for example, SMART voice-operated lightbulbs were seen as useful predominantly for immobile individuals. Arguably, however, some of these technologies could help fulfill the needs of any person irrespective of age (eg, various indoor sensors and internet-based assistants to aid in activities of daily living). It should be noted that this assessment may reflect the inability of the generally healthy and high-functioning older adults in this study to accept the eventual probability of needing different forms of specialized care in later years (an analogous process has been documented with respect to perceptions of frailty) [31]. This further underscores the need to introduce and explain potential SMART technologies to older adults and provide specific relatable scenarios where they could be useful for them personally.

Older adults and experts were rather consistent in what should already be commonplace or what are the good examples of SMART technologies for older adults: fall detectors, SOS buttons, smart beds, some smart tools of daily living, etc. One technology that experts saw a great benefit in, but older adults did not find very useful and voiced concerns over, was social or interactive robots. This sentiment was also echoed by professional caregivers who saw these technologies as potentially displacing important needs of older adults (ie, need for personal contact). This was a surprising finding, given the literature that generally emphasizes the advantages of social robots and supports their social benefits [32].

Taken altogether, the views expressed by professional caregivers, older adults, and aging experts indicated that once objective barriers such as cost and accessibility are successfully confronted, a key prerequisite to higher adoption of SMART technologies in older adult care is the overall increase in digital literacy both at the caregiver and older adult level. Increasing knowledge of the benefits of digital technologies that can be linked to the specific needs of older adults and their caregivers should be of utmost importance. These changes will partly occur naturally, without significant efforts from the outside, because of the aging of the population for whom it is natural to use SMART technologies in everyday life. Nevertheless, this process could be potentially accelerated in several ways. For instance, “safe spaces” could be created for older adults and their caregivers to try out technologies, gain personal experience, and subsequently make an informed decision on purchase and use. Similarly, potential users of SMART technologies for older adult care would benefit from an overview of the technologies available in the market, ideally linked to the needs of older adults and caregivers that could be fulfilled by using these technologies. In addition, educational programs directed both at caregivers and older adults would be useful tools for improving digital literacy, attitudes toward technology, and eventual use of technology [33].

Our case study also shows that it is important to find and communicate “selling points” of SMART technologies to the facilities and caregivers and that the original use case and functionality may not prove to be the most useful for the customer. For example, when the ANUME smart bed system was first considered by SeneCura, the application partner, its most desired feature was considered to be the monitoring of vital signs while in bed. However, the most recent iteration of the technology does not even include real-time data on vital signs; instead, information on bed exits has been prioritized by the caring staff, and the positioning incident report has been adopted as the primary tool by the management staff. These gradually transforming and emerging needs would not have been captured without an ongoing shared dialog between the researchers, the technology developers, and the care facility staff and management. In addition, SeneCura communicated that this iterative reciprocal process helped overcome the initial skepticism of some of the staff toward the technology and made them use the technology more (ie, their needs were “heard”).

### Strengths and Limitations

The main strengths of this study lie in incorporating multiple perspectives on the use and adoption of SMART technologies for improving care of older adults and presenting a concrete example of technology implementation in a caregiving context, which is rarely done in one comprehensive study. Nonetheless, this study has several limitations. First, it was cross-sectional and thus limited in terms of causality. Second, the study included perspectives of only a small sample of professional caregivers and a selective group of older adults (attendees of the V-U3A). These more technologically savvy older adults could have a more positive view of technology than the general older adult population. Similarly, the comparatively more “negative” evaluation of older adults’ abilities by professional caregivers could reflect the specific clientele they serve (ie, older adults in older adult homes or in need of care). Third, despite the successful example of collaboration with an industry partner, SeneCura, it must be noted that the management of SeneCura was very receptive to innovations to begin with, which may have influenced the findings of this study. According to the Diffusion of Innovations theory [34,35], innovators and early adopters are more likely to embrace new technologies compared with the majority of the population. In the context of a research study, if a care provider falls into the category of innovators or early adopters and exhibits a high level of enthusiasm and openness toward technology, their adoption behavior may not accurately represent the attitudes and behaviors of the broader population. To avoid this potential pitfall in future research studies, several strategies such as diverse participant recruitment (ie, a broad range of care providers with varied levels of technology receptiveness); separate data analyses for different segments of care providers (ie, based on their technology adoption profiles); or contextual exploration (organizational culture, resource availability, training opportunities, etc) can be used.

### Future Directions

Regarding the driving force of adoption of SMART technologies, with significant barriers to adoption at multiple levels, it is unrealistic to expect that caregivers or older adults would be the primary or sole actors in this process. Even if SMART technology product owners, developers, and designers strive to develop products that are usable for older adults with adjusted interfaces and operation where necessary (a need that was underlined by the experts in this study), this by itself will likely not be sufficient to overcome the abovementioned barriers and convince the potentially reluctant end users. Synergies must be created among efforts on the side of the researchers, caregivers, adopting settings (facilities), and potential business partners to ensure successful adoption of SMART technologies and their long-term use in care for older adults. Experts explicitly pointed at a “missing link” represented by a facilitator that would assist with the implementation of the technology as well as technical support in the process of use. If SMART technologies for older adults show the promise of profit, the adoption might be naturally facilitated by interested business parties. However, with institutionalized care for older adults being a difficult segment to navigate (ie, many regulations related to health services, slow-moving) and home care being an underfunded and underrated segment, the risk of limited interest of business partners to enter these market segments, develop specifically for them, and additionally take on the difficult facilitating and support role is evident. This is where nonprofit organizations and government-funded agencies might prove highly beneficial in facilitating the connection between researchers, business partners and end users, be it older adults, care facilities, or individual caregivers; providing educational programs on technology; and creating the “safe spaces” to gain a firsthand experience with the technology. Future research studies on this topic should incorporate longitudinal designs to track technology adoption over time and ideally in combination with a mixed methods approach to capture not only the statistical trends but also the richness of the underlying motivations, barriers, and experiences of different stakeholders to provide a more comprehensive understanding of technology adoption. Theoretical frameworks related to the diffusion or spread of technologies (such as the Diffusions of Innovations theory) can help provide a theoretical foundation for such work.

## Appendix

Multimedia Appendix 1 Data collection instruments.

Multimedia Appendix 2 Evaluation of technologies by older adults.

## Abbreviations

V-U3A: Virtual University of the Third Age

## Data Availability

Data used in this study are available upon request from the first author.
