# Supplementary material for: Multiple Perspectives on the Adoption of SMART Technologies for Improving Care of Older People: Mixed Methods Study
Source: J Med Internet Res. 2024 Feb 7;26:e45492. doi: 10.2196/45492 (PMC10882473; doi:10.2196/45492)
Supplement: Multimedia Appendix 1 [file jmir_v26i1e45492_app1.docx]

# Methods

### Study 1: Perspectives of Professional Caregivers

**Purposive Sampling Strategy**

Recruitment for Study 1 was carried out through selected cooperating facilities and through chain referral. The recruitment process used a purposive sampling strategy [1]. It was important for us to select a heterogeneous group of caregivers on the basis of the dominant work activity performed in their job positions. This approach to the selection of respondents helps ensure a more comprehensive view of the use of smart technologies in elderly care and provide information not burdened by a limited or homogenous view of the issue because of similarities in work experience. Thus, our basic goal in selecting respondents was to reach a wider spectrum of care worker positions and the resulting sample included five social workers (in charge of most non-medical care of the elderly), four managers (head of direct care, head of health care, head social worker, head of the social services department), two health workers (general nurse, station nurse in home care) and one caregiver.

**Interview Guide**

Pilot interviews showed that it was generally difficult for the respondents to envision what SMART technology means in care for older adults and it was difficult to discuss the possible use and benefits of such technologies without having previous personal experience with it. Thus, after the first pilot interviews (5), the "interview guide" was slightly modified to provide more space for the discussion of concrete examples of technologies. To that end, the guide was supplemented with a PowerPoint presentation in which 19 different technologies/products were presented (photos and a short description/explanation of its use). Respondents then commented on their usability in the context of their job position and caring experience. The adjustment implemented in this way had a positive effect both on the quality of the interviews and the depth of the information obtained.

The final version of the "interview guide" consisted of two parts. The first part related to general information about the respondent and his work experience and was structured into three thematic units: attitudes and evaluation of work (2 questions); context (2 questions); communication and interaction with family (1 questions). The second part was focused on the discussion of specific smart technologies with support of the PowerPoint presentation. During the presentation, the respondents evaluated the usability of the given technologies in the context of their job positions and experience, barriers, risks and opportunities associated with them.

| **Personal Information** | | **ID_____________** | |
| --- | --- | --- | --- |
| **Interview start:** | | **Interview end:** | |
| Age |  | Work Position |  |
| Gender | 1. Female    2. Male |  |  |
| Education | 1. Elementary (even if not completed) 2. Secondary/vocational (without high school diploma) 3. Secondary with high school diploma 4. University degree and higher |  |  |
|  |  | Previous work experience (relevant to caregiving/health care) |  |
|  |  | How long have you worked in the area of older adult care? |  |

**Attitudes and job evaluation**

1. What do you consider to be the most satisfying aspects of your work?
2. What makes your job difficult and what could make it easier?

- As part of daily tasks (e.g., client positioning, activation, night checks, etc.)
- In the longer term

**Context**

1. Which clients do you care for?

- How different are the clients? Do clients tend to be similar or do you need a slightly different approach to each?
  - Describe age group, degree of dependency (autonomy, self-care), health status

1. What could make communication with family/loved ones easier?
2. What does the client intake process look like? (QUESTION FOR SOCIAL WORKERS ONLY)
3. What could make the transition process from home to residential care easier?

**The role of technology**

1. Now I would like to ask you about the use of MODERN TECHNOLOGY to facilitate care, whether you already use some technology or whether you can imagine it being useful.
2. We are now interested in your perceptions of the different tools that are used to deliver care. (PPT PRESENTATION FOLLOWS NEXT)

| 1 |  | 5 |  | 9 |  | 13 |  | 17 |  |
| --- | --- | --- | --- | --- | --- | --- | --- | --- | --- |
| 2 |  | 6 |  | 10 |  | 14 |  | 18 |  |
| 3 |  | 7 |  | 11 |  | 15 |  | 19 |  |
| 4 |  | 8 |  | 12 |  | 16 |  | 20 |  |

1. What obstacles, risks, pitfalls do you see in engaging these technologies? INQUIRE ABOUT BARRIERS DURING THE CONVERSATION FOR THE DIFFERENT TYPES OF TECHNOLOGIES DISCUSSED.
2. Can you think of any other technologies that can be used in care or are you interested in any of our examples?

**References:**

1. Palinkas LA, Horwitz SM, Green CA, et al. (2015) Purposeful sampling for qualitative data collection and analysis in mixed method implementation research. Administration and Policy in Mental Health and Mental Health Services Research 42(5): 533–544.

### Study 2: Perspectives of Older Adults

**Online Survey**

The online survey was administered in Qualtrics. Prior to deployment, the survey was pilot tested internally by the research team to detect technical problems, content issues and ensure proper flow. The survey was voluntary and “open”. The survey was distributed via email with a single link. Access to the survey was also made possible through the project website. No renumeration was offered for participation.

The survey was presented across 30 pages with a maximum of 4 questions presented on one page. Individual technologies (n=21) were presented in separate pages. Each technology was evaluated by one “rating” question and one open-ended question. Respondents were allowed to go back to previous questions and could save the survey and finish completing it later (within 1 week).

First of all, we would like to know something about you.

What is your gender?

- Man (1)
- Woman (2)

Please enter your year of birth.

________________________________________________________________

What is your marital status?

- single (1)
- married (2)
- cohabitation (3)
- divorced (4)
- widower/widow (5)

Your highest level of education

- Basic (and incomplete) (1)
- High school diploma, vocational secondary without matriculation (2)
- Secondary education (general and vocational) (3)
- Higher Vocational (post-secondary, DiS) (4)
- University and higher (Bc., Mgr., Ing., Ph.D....) (5)

Your social status and current economic activity. Select/tick all that apply.

- In employment (1)
- Private entrepreneur/ self-employed/freelance (2)
- At home (including parental leave) (3)
- Permanently caring for a loved one (4)
- Retired (old age/disability) (5)
- Unemployed (6)
- Other (7) __________________________________________________

How many **hours a week on average** do you devote to your work activities? Please indicate the number:

________________________________________________________________

How would you rate your family's economic situation?

- Below average (1)
- Rather below average (2)
- Average (3)
- Rather above average (4)
- Above average (5)

How would you rate your skills in using IT technology (e.g. computer, smartphone, tablet)?

- Beginner 1 (1)
- 2 (2)
- 3 (3)
- 4 (4)
- 5 (5)
- 6 (6)
- Expert 7 (7)

In the following section, we are interested in your feedback on various technologies that are currently being used (or developed) for the target group of seniors. Please provide us with your assessment of these technologies. There are no right or wrong answers, we would like to hear your opinion. Thank you.

**The RFID chip** allows to track the movement of people; to read the recorded information (name, health status, indicated medication, etc.) using a reader. RFID chips can take the form of not only bracelets, but also various pendants or can be sewn into clothing.

**The barcode reader** allows you to read the information using the barcode and transfer it to the system (e.g. the performance recorded in real time, etc.). These readers are used in the provision of care (e.g. by a care service) to record the actions (services) performed or the administration of medication

**A special smart spoon** enables the absorption of hand tremors (e.g. in Parkinson's disease) and thus supports the independence of the elderly person.

**Smart bulbs** allow you to control (turn off, turn on, dim) individual lights by voice

**Systems such as "WITRACK"** allow tracking the movement of a person in a space using sensors placed in the space (e.g. on the wall, etc.). It can detect falls, turn off/on household appliances. It can also track movement in a different room than the sensors are located.

**Wearable sensors** such as SOS buttons detect falls and can summon help. The senior must wear it on his/her person (e.g. on the neck).

**Wearable wristbands** (such as Fitbit) allow you to track certain physical activities (steps, distance, etc.), assess sleep and alert you to sedentary behaviour.

**The interactive table** "SenTable" can be used for entertainment, but also for games designed for memory training or reminiscence therapy.

**A tablet** can be used not only for entertainment, but also to communicate with family, record and search for information.

**A smartphone** can be used not only for entertainment, but also for communicating with family, recording and searching for information.

**The smart alarm clock** is voice-controlled and can play music (or connect a mobile phone to recharge).

**The smart bed** "Anume" consists of sensors placed under the mattress and monitors breathing, heart rate. The system can generate clinical reports and display notifications and trends indicating risk conditions and possible diseases (coronary heart disease, rhythm disorders - arrhythmias, fibrillation, valve disorders, myocardial infarction, asthma, apnea, pneumonia). It does not require any manipulation by the client.

**The smart cup** monitors the amount of liquid you drink. It can alert you when a drink is too hot.

**The smart medication dispenser** allows you to set individual medication dosages. It warns visually and audibly.

**A mobile app** on your phone or tablet that allows you to track and check the completion of tasks. Completion can be seen by all people logged into the app.

**Interactive robots** can answer questions, search for information, sing and dance. As it develops, it should be able to carry on a normal conversation.

**Sensors in the room** can sense temperature, light intensity, detect smoke, etc.

**The smart non-contact thermometer** allows non-contact measurement of body temperature, surface temperature or object temperature.

**The smart scale** offers measurements of body weight, body fat, water, etc. It can be connected to a mobile app.

**Virtual reality** allows you to experience a simulated environment through special 3D glasses. For seniors, it is used, for example, as a way to virtually "travel" or visit sights and other regions.

**The virtual assistant,** which is connected to the internet and controlled by voice, helps to search for information, can remind you to take your medication or order food or transport.

***For each technology, a description and photograph was presented along with two follow-up questions:***

Reflect on the challenges of old age and rate how useful you consider the tool/technology to be for seniors/people of retirement age.

- Minimally useful 1 (1)
- 2 (2)
- 3 (3)
- 4 (4)
- 5 (5)
- 6 (6)
- 7 (7)
- 8 (8)
- 9 (9)
- Maximally useful 10 (10)
- I don't know (11)

If you were to use the tool/technology, would you have any concerns? Please briefly describe:

________________________________________________________________

________________________________________________________________

________________________________________________________________

- - - - - - - - - - - - - - - - - - - - - - - - - - - - - - - - - - - - - - - - - - - - - - - - - - - - - - - - - - - - - - - - - -

Is there anything else you would like to tell us about the use of technology for the elderly? Please elaborate:

________________________________________________________________

________________________________________________________________

________________________________________________________________

The last section of the questionnaire asks whether you are caring for another person over 65. Please answer the following questions.

Care Do you provide care to anyone over 65?

- YES (1)
- NO (2)

Skip To: End of Block If Are you providing care to someone over 65? = NO

You said you provide care for an older person (over 65). Who are you caring for? (which older person in relation to you)

- Mother (1)
- Father (2)
- Thane (3)
- Mother-in-law (4)
- Other family member (specify which) (5) __________________________________________________
- Other person (specify) (6) __________________________________________________

Now we are interested in some information about the person you are caring for. If you care for more than one person, please relate your answers to the primary person you care for (i.e. the person you spend most of your time caring for).

What is the general health of the person you care for?

- Very good (1)
- Rather good (2)
- Neither good / nor bad (3)
- Rather bad (4)
- Very bad (5)

How is the person you are caring for?

|  | Completely  1 (1) | 2 (2) | 3 (3) | 4 (4) | 5 (5) |
| --- | --- | --- | --- | --- | --- |
| Mobile (1) |  |  |  |  |  |
| Self-sufficient (2) |  |  |  |  |  |

Has the person you care for been diagnosed with dementia (of any type)?

- YES (1)
- NO (2)

How often do you care for this person?

- Continuous (all day and night) (1)
- All day (but not at night) (2)
- Every day, but not all the time (3)
- 3-5 x weekly (4)
- 2 x weekly (5)
- Several times a month, but not every week (6)
- Less often than once a month (7)

Do you receive Attendance Allowance?

- YES (1)
- NO (2)

Do you share the care of an older person with someone else?

- YES (1)
- NO (2)

Skip To: End of Block If Do you share the care of an older person with someone else? = NO

How many other people do you share care with?

________________________________________________________________

How much of the care would you say is up to you?

|  | 0 | 10 | 20 | 30 | 40 | 50 | 60 | 70 | 80 | 90 | 100 |
| --- | --- | --- | --- | --- | --- | --- | --- | --- | --- | --- | --- |

| Please indicate the percentage (%) () | 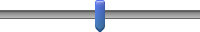 |
| --- | --- |

In relation to the care you provide to another person over 65, is there anything else you would like to tell us about the use of technology in the context of elderly care? Please elaborate:

________________________________________________________________

________________________________________________________________

________________________________________________________________

________________________________________________________________

### Study 3: Perspectives of Experts on Aging

**Email Interview**

With respect to time-constraints of the respondents, the interview was constructed to be very brief with 5 open-ended questions on SMART/modern technology for the elderly. The questions covered the following areas: (i) opportunities of SMART/modern technologies for seniors; (ii) barriers of SMART/modern technologies for seniors, (iii) good practice examples, (iv) technologies that should be commonplace in senior care, (v) recommendations to stakeholders researching and implementing technologies for seniors.

**Use of modern/smart technologies in older adult care**

Expert interview

**Directions:** please answer the following questions taking into account the definition below. There is no word limit for responses, and we welcome more extensive responses based on your ability and desire to comment on the topic.

**Definition:**

The following questions relate to, but are not limited to, the application of modern/smart technologies, which for the purposes of this interview we view broadly. By modern/smart technologies, we mean digital and other technologies that enable at least some of the following functionalities:

- health monitoring (e.g., vital signs, movement, health, etc.)
- data evaluation (analysis) and reporting
- Internet connectivity/remote data management
- interaction/communication with the user
- interactive and autonomous functioning (e.g., with artificial intelligence elements)
- connection to other devices/technologies

**Questions:**

1. What opportunities do you see in the use of modern/smart technologies in older adult care in the Czech Republic?

*Please answer here...*

1. What do you see as the most significant barriers to the use of modern/smart technologies in older adult care in the Czech Republic? Alternatively, how do you think these barriers could be eliminated?

*Please answer here...*

1. Have you come across any examples of good practice in the use of modern/smart technologies in older adult care in the Czech Republic or abroad? If yes, could you describe it?

*Please answer here...*

1. What modern/smart technologies do you think should already be "commonplace" in older adult care (even though this may not be the case in the Czech context)?

*Please answer here...*

1. If you could give a message or direct recommendation to people/companies involved in the development and use of smart technologies to facilitate older adult care, what would it be?

*Please reply here...*

Please use the remaining space to make any other comments you feel are relevant to the issue:

*Please reply here...*
