# Supplementary material for: Multiple Perspectives on the Adoption of SMART Technologies for Improving Care of Older People: Mixed Methods Study
Source: J Med Internet Res. 2024 Feb 7;26:e45492. doi: 10.2196/45492 (PMC10882473; doi:10.2196/45492)
Supplement: Multimedia Appendix 2 [file jmir_v26i1e45492_app2.docx]

| In the following section, we are interested in your feedback on various technologies that are currently being used (or developed) for the target group of seniors. Please provide us with your assessment of these technologies. There are no right or wrong answers, we would like to hear your opinion. Thank you. | | | | |
| --- | --- | --- | --- | --- |
| **Technology - description** | **Think about the challenges of old age and rate the extent to which you consider the tool/technology to be useful for seniors/people of retirement age.** | | | **If you were to use the tool/technology, would you have any concerns? Please briefly describe:** |
|  | Mean | Standard Deviation | Number of responses | *Examples of open-ended responses* |
| **Wearable sensors such as SOS buttons detect falls and can summon help. The senior must wear it on his/her person (e.g. around the neck).** | 8.88 | 1.75 | 310 | Number of comments: 219  *"1/ loss of privacy 2/ I see the problem in having to wear the sensor all the time - frequent falls are in the bathroom for example..."*  *"Still should detect where I am. worried about replacing and buying batteries. Need sim cards and not suitable tariffs."*  *"I certainly wouldn't worry, certainly easier for a senior to use this device than a mobile phone to call for help."* |
| **A tablet** can be used not only for entertainment, but also to communicate with family, to record and retrieve information. | 8.78 | 1.77 | 314 | Number of comments:220  *"Nowadays with more family distance I appreciate the ability to make phone calls and see each other, the ability to stay connected with friends, even reading messages is easier with the ability to enlarge the font."*  *"A device that will actually work without problems will be expensive, internet services are starting to charge and so most useful things and programs will be unavailable. The ordinary pensioner needs someone who, if he gets stuck in any step, will help him, advise him."*  *"There is no substitute for personal contact, but if contact is not possible or only limited, then a great thing. Of course, if the senior knows how to use it, it's also a great tool to get information, entertainment, ....."* |
| **A smartphone** can be used for entertainment and communicating with family, recording and searching for information. | 8.73 | 2.05 | 313 | Number of comments: 229  *"I own a smartphone but don't know how to use it fully. Operators don't give instruction manuals, they don't teach kids...not enough courses for seniors to increase literacy in using this technology."*  *"Not a concern, I just think sometimes smartphones are too over the top for older people. And then they annoy more than they help. But for those who can it's a good thing. I personally find them too complicated."*  *"I don't take a step without it and I take full advantage of the capabilities I'm able to use."* |
| **The smart bed** "Anume" consists of sensors placed under the mattress and monitors breathing, heart rate. The system can generate clinical reports and display notifications and trends indicating risk conditions and possible diseases (coronary heart disease. rhythm disorders - arrhythmias. fibrillation. valve disorders. myocardial infarction. asthma. apnea. pneumonia). Requires no manipulation by the client. | 8.65 | 1.98 | 291 | Number of comments: 209  *"I don't need such a bed yet thankfully, otherwise I would also be interested in the price and any contribution from the health insurance company."*  *"There could be constant monitoring of heart rate and breathing information and subsequent panic. It definitely depends on the health of the senior."*  *"This seems good (almost surreal) for the caregiver. Is there really such a thing?"* |
| **The smart non-contact thermometer** allows non-contact measurement of body temperature. temperature of surfaces or objects. | 8.59 | 1.96 | 301 | Number of comments: 186  *"Thermometers would be excellent if they worked properly. I've had a lot of bad experiences with them (even with different qualities)."*  *"I have no concerns, after all, similar thermometers are already in use, see the measurement of patients in the waiting room during the covid period..."*  *"I don't want to, the daughter of a nurse trusts more classical ones. The non-contact ones are more malfunctioning."* |
| **A special smart spoon** allows the absorption of hand tremors (e.g. in Parkinson's disease) to support the independence of the elderly person. | 8.42 | 2.21 | 295 | Number of comments: 236  *"As long as the spoon isn't heavy and the food doesn't slip out of the spoon.."*  *"I was excited about this, considering my mom suffered from Parkinson's, it would help her a lot mentally.."*  *"I find it unnecessary. Either the senior "somehow" eats with a normal spoon or the caregiver feeds him....."* |
| **The interactive table** "SenTable" can be used for entertainment. The "SenTable" can also be used for games designed for memory training or reminiscence therapy. | 8.07 | 2.18 | 297 | Number of comments: 215  "*They should be intertwined with personal memories of the individual's youth and experiences. Anyone who hasn't used a tablet or touchscreen phone won't even be able to click."*  *"I think active communication with a live person rather than a tablet is more important in the elderly. But in times of need, maybe it is. A drowning man grasps at straws, so I'd rather have a whiteboard than nothing..."*  *"This is useless! It can be replaced with board games on the table, which will force you to practice your touch, hands, observation to keep it from falling, etc."* |
| **A smart pill dispenser** allows you to set individual medication dosages. It warns visually and audibly. | 7.95 | 2.46 | 290 | Number of comments: 202  *"Concerns - program the smart dispenser correctly"*  *"concerns I would not have. However, those dispensers where the medication is divided into morning-noon-evening seem clearer to me at first."*  *"Unnecessary for me. I know from experience that for some seniors, dispensing medication is almost a ritual."* |
| **Smart lightbulbs** allow you to control (turn off. turn on. dim) individual lights by voice. | 7.87 | 2.45 | 300 | Number of comments: 230  *"No worries, it may be a good thing for a certain group, I'm just thinking of a situation where a person is in a state of not controlling their voice well, that it would light up when they don't even want it to, they would just make the required sound without realizing it and it would light up when they really don't even want it to."*  *"many seniors don't have that strong of a voice, will a light bulb respond to a weak voice? I know from personal experience that seniors prefer light over darkness."*  *"I'm not familiar with this device yet and can't imagine its benefits."* |
| **Systems such as "WITRACK**" allow tracking a person's movement in space using sensors placed in the space (e.g. on walls, etc.). It can detect falls. turn off/on household appliances. It can also track movement in a different room than the sensors are located. | 7.83 | 2.41 | 276 | Number of comments: 224  *"Yes. A great invasion of privacy. Just an SOS button."*  *"It's probably the "music of the future", again the danger of misusing the data for a different purpose than intended."*  *"To feel like I'm constantly being monitored would probably make me nervous. On the other hand - for the medical condition for which this sophisticated system is developed, it is a chance to be able to stay in my own environment with some sense of security."* |
| **Environmental sensors** in the room can sense temperature. light intensity. detect smoke, etc. | 7.77 | 2.50 | 283 | Number of comments: 185  *"Concerns to make sure everything works smoothly and to be able to rely 100% on all the conveniences."*  *"Some features facilitating independent thinking seem "counterproductive""*  *"Concerns no, I would rather have peace of mind when I move away from the person I am caring for."* |
| **A mobile app** on a phone or tablet allows you to monitor and check the completion of tasks. Completion can be seen by all persons logged into the app. | 7.11 | 2.79 | 226 | Number of comments: 186  *"I suppose the apps can be used/used in facilities where the sick, infirm are cared for. It will definitely make the work of the staff in these facilities easier and more helpful."*  *"Maximum useful to the health care industry. Especially now in the covid era when staff is overloaded and when they can easily overlook or forget something. The question is who will have the time, the tablet to feed the information."*  *"Just don't forget to look at the phone."* |
| **Wearable wristbands** (such as Fitbit) allow tracking of certain physical activities (steps. distance etc.). assess sleep and alert on sedentary behavior. | 7.10 | 2.68 | 305 | Number of comments: 223  *"I don't find that this technology solves the problems of old age, but it may be interesting for some to watch."*  *"The device in question is considerably very small and reading the stored readings is difficult. And connecting to another device a smartphone or PC is probably not manageable for these people."*  *"I've had it before, it just annoyed me. I was resting after a 10km walk and my watch was telling me I should get up again. I wanted to step on it. I haven't worn it since."* |
| **A virtual assistant** which is connected to the Internet and controlled by voice. helps to look up information, can remind you to take medication or order food or transportation. | 7.04 | 2.66 | 244 | Number of comments: 176  *"Learning how to operate this technology."*  *"Another informative thing, but not so much needed, given the age, ease and simplicity of operating the various gadgets is important."*  *"I don't think an old person would know how to operate it. And if he does, then he doesn't need it."* |
| **The smart cup** monitors the amount of liquid you drink. It can alert you when a drink is too hot. | 6.69 | 2.91 | 284 | Number of comments: 200  *"No, but it's more of a caregiver's aid. I'm sure most people would prefer their favorite mug."*  *"Unnecessary. If I'm still considering it, I don't drink boiling. If someone is dependent on the care of another person - hopefully no one will serve them a brew."*  *"So I would use this mug with enthusiasm because I forget to drink during the day."* |
| **Smart scale** offers measurement of body weight, body fat, water etc. It can be linked to a mobile app. | 6.65 | 2.77 | 292 | Number of comments: 178  *"I find this scale unnecessary, I have it and only use it to measure my weight."*  *"Such a scale is only for a "young" pensioner, normally mobile, who can see the readings at his feet in addition from his height."*  *"It may not be bad for someone, but it seems a bit unnecessary, but how about inventing a scale for the immobile and recumbent?"* |
| **RFID chip** allows tracking the movement of people; using the reader to read the recorded information (name. health status. indicated medication etc.). RFID chips can take the form of not only wristbands. but also various pendants or can be sewn into clothing. | 6.50 | 2.62 | 301 | Number of comments: 278  *"In general I find such a tool maximally useful, but the degree of usefulness obviously depends on the specific living situation, health and self-sufficiency Concerns? Anyway, I would feel a loss of privacy."*  *"Great, especially for people who live alone. I would assume that the system would be protected so that it would not be abused, willingly or unwillingly."*  *"Only useful in the event of dementia, loss of orientation, or for adrenaline sports in the event of some sort of accident or avalanche... Otherwise, I'm an opponent of any kind of human tracking."* |
| **The barcode reader** allows information to be read using the barcode and transferred to the system (e.g., the performance recorded in real-time etc.). These readers are used in the provision of care (e.g., by a care service) to record the actions (services) performed or the administration of medication. | 6.36 | 2.86 | 285 | Number of comments: 238  *"I wouldn't see any cause for concern here. As a former nurse, I would consider such a tool to be a great help."*  *"all we need is paper and pencil, when the power goes out it will be painted.... I'm not a pessimist, but this doesn't bother me."*  *"but I'd feel a bit like a robot being scanned for information."* |
| **The smart alarm clock** is set by voice. It can play music (or a mobile phone can be connected to recharge). | 6.29 | 2.78 | 282 | Number of comments: 206  *"Concerns I wouldn't have, but if I have a smart phone, I would consider this device redundant."*  *"Concerned about laborious maintenance (perpetual reconfiguration) when in use."*  *"An invaluable aid for the visually impaired. It will also help the elderly."* |
| **Virtual reality** allows you to experience a simulated environment through special 3D glasses. For seniors, it is used, for example, as a way to virtually "travel" or visit sights and other regions. | 6.20 | 2.98 | 260 | Number of comments: 179  *"Real old age no longer needs such things, peace of mind is preferred to the unnecessary excitement of modern life. I would see it advantageous in conversations with relatives, family."*  *"Side effects such as nausea, headaches in sensitive persons, tried."*  *"I wouldn't have any concerns, I'd be more interested in whether I could use the glasses with my impaired vision /retinal disorders, cataracts/"* |
| **Interactive robots** can answer questions, search for information, sing, dance. As it develops, it should be able to carry on a normal conversation. | 4.55 | 2.91 | 238 | Number of comments: 191  *"Unnecessary toy. Then I guess no senior citizen desires one. Needs human contact."*  *"An old person needs personal contact not a robot."*  *"for the next generation of retirees - for those who don't have children or friends of their own"* |
|  | **1. Is there anything else you would like to tell us about the use of technology for the elderly? Please elaborate:**  **2. In relation to the care you provide to another person over 65, is there anything else you would like to tell us**  **about the use of technology in the context of elder care? Please elaborate:** | | | |
|  | Number of comments: 195/103  *"The older a senior gets, the more scared he is of new technology, everything is new to him. He's afraid it will break, shut down, etc. I myself would like to be able to use new technology the way my children and grandchildren can. I try, but it doesn't work as well as I would like."*  *"It's not only the old person who needs love, caresses, company. No technology can replace that. Rather, technology will help to take better care of the person and to have time for personal contact with them!!!! But I fear that personal contact will be even more limited. That's just prolonging life. Life????!!!...... It gives me the shivers."*  *"Doing things for them that are clever but not too complicated. And ones that can be used not only by the elderly, but maybe for younger disabled people in a home environment, so that they can handle it themselves and not be dependent on someone else to use it again."* | | | |
